# The Prevalence of Dysphagia in Children with Laryngomalacia Pre and Postsupraglottoplasty: A Systematic Review with Meta-Analysis

**DOI:** 10.1055/s-0042-1755309

**Published:** 2024-02-05

**Authors:** Eduarda Pinto Rossoni, Vanessa Souza Gigoski de Miranda, Lisiane De Rosa Barbosa

**Affiliations:** 1 Universidade Federal de Ciências da Saúde de Porto Alegre (UFCSPA), Porto Alegre, RS, Brazil; 2 Universidade Federal do Rio Grande do Sul (UFRGS), Porto Alegre, RS, Brazil; 3 Department of Speech Therapy, Universidade Federal de Ciências da Saúde de Porto Alegre (UFCSPA), Porto Alegre, RS, Brazil; 4 Irmandade of Santa Casa de Misericórdia, Hospital da Criança Santo Antônio, Porto Alegre, RS, Brazil

**Keywords:** infant, newborn, deglutition, deglutition disorders, laryngomalacia, aspiration

## Abstract

**Introduction** Laryngomalacia is the most common congenital laryngeal alteration, with spontaneous resolution in most cases. However, in the face of more severe presentations of the disease, it is necessary to perform supraglottoplasty surgery. Studies have been dedicated to researching changes in swallowing in children with laryngomalacia before and after surgical intervention.

**Objectives** To identify the prevalence of oropharyngeal dysphagia in children with pre and postsupraglottoplasty laryngomalacia.

**Data Synthesis** A search strategy was developed with terms and entreterms to designate a population *pediatric with laryngomalacia* , exposure *supraglottoplasty* , and outcome *frequency of dysphagia* , adapted to the requirements of the main databases in the health area. The analysis of the records found was performed by two independent examiners and, in the end, 6 articles were included in the study. The articles found enabled a sample of 330 children with laryngomalacia, 311 of whom underwent supraglottoplasty. Among the included studies, 5 were grouped and meta-analyzed. After supraglottoplasty surgery, a 59% reduction in the prevalence of oropharyngeal dysphagia was identified, with high heterogeneity I ^2^ = 93%.

**Conclusion** Despite the heterogeneity of the sample, the supraglottoplasty procedure significantly reduces the prevalence of dysphagia in children with laryngomalacia.

## Introduction

Laryngomalacia (LM) is the most common congenital laryngeal alteration, 1 2 3 4 being responsible for 70% of cases 4 of stridor in babies, 1 3 4 5 a symptom that is characteristic of the disorder. The stridor consists of a high-pitched breathing sound that signals an obstruction in the airways, 2 6 7 more precisely at the laryngeal and/or tracheobronchial levels. 8 Airway obstruction results from the collapse of the supraglottic region, 2 7 due to excess mucosa and alteration and/or reduction in laryngeal tone. 2

Stridor characteristics such as intensity and frequency, among others, will depend on the extent of the obstruction and caliber of the affected airways, as well as on the respiratory effort and alteration of the air flow speed that may be being used at the moment. 5 In addition, stridor usually worsens with feeding, 8 supine positioning (5.8), agitation 7 and may present as a single symptom or with other symptoms, such as respiratory effort, tachypnea, and chest retraction, among others. 5 6 8

The diagnosis of LM takes into account the patient's clinical history, but it is confirmed, most of the time, with a flexible fiberoptic laryngoscopy exam, which investigates the dynamic movement of the laryngeal structures during breathing, performed with the patient awake 2 5 7 8 or under sedation in spontaneous breathing. The main findings of the examination are prolapse of supra-arytenoid tissue during inspiration, epiglottis with an omega or retroflex shape, shortened aryepiglottic folds, redundant arytenoid mucosa, poor visualization of the vocal folds, and edema of the posterior glottis region. 2 3 6 7 8

The symptoms of LM usually begin to appear in the 2 ^nd^ week of life, but, in most cases, they resolve spontaneously with little or no treatment until 18 to 24 months of age. 1 2 8 The spontaneous resolution of cases often requires, in addition to monitoring the patient, changes in their diet and specific treatment for gastroesophageal reflux (GER). 2 4 Between 10 and 20% of cases require surgical intervention, 1 2 7 which can also vary according to the complexity of the service studied. 7

Surgical treatment of LM is determined by the severity of the patient's symptoms, 1 2 7 with absolute indication for patients who present conditions such as cor pulmonale and/or pulmonary, pulmonary hypertension, pectus excavatum, hypoxia, and ventilatory failure, among others respiratory compromises that pose risks to the patient's life. Other indications considered relative are weight loss associated with feeding difficulties, laryngotracheal aspiration with recurrent pneumonia, and obstructive sleep apnea. 2 7 The endoscopic procedure developed to alter the anatomy of the supraglottic area preventing collapse and obstruction of the region of the larynx is called supraglottoplasty (SGP). 9

Laryngomalacia can also be classified according to its severity as mild, moderate, and severe, based on the association of symptoms in the diet and airway obstruction. Mild LM usually has no consequences and can be managed through observation and monitoring, without interventions. Moderate LM has symptoms related to diet, but it is still possible to circumvent them through dietary changes and acid suppression in cases of gastroesophageal reflux (GER). The severe form of the disorder, on the other hand, requires surgical intervention through SGP. It is believed that between 10 and 20% of patients initially present with severe LM. 7 In any of the degrees of severity, it is necessary to carry out continuous monitoring of patients and observation of their symptoms, as there may be a progression in the classification of cases. 2 7

Difficulties in feeding, dysphagia, aspiration, and silent aspiration are highly prevalent in LM. 9 10 11 As it is an anomaly that alters the breathing pattern, it ends up interfering in the coordination between sucking, swallowing, and breathing, functions that are so important, especially for the act of eating safely. The main consequences of this incoordination are pulmonary and nutritional impairment. 12 A possible cause for feeding difficulties and swallowing disorders in patients with LM is the decreased sensitivity of the laryngeal region due to reflux of gastric contents, a comorbidity present in most patients. 13

The true prevalence of dysphagia and laryngotracheal aspiration in infants with LM is still unknown 10 11 due to the lack of regularity in the objective assessment of swallowing in these patients. 10 14 Such an evaluation presents a greater sensitivity for the detection of aspiration, mainly of silent aspiration. The tools for clinical evaluation of swallowing are based, mainly, on signs and symptoms suggestive of dysphagia, and in silent aspiration, these signs are not evident. 11

Silent aspiration is classified as the passage of food, liquids, medications and other substances towards the lower airways, below the vocal folds, without triggering the cough reflex. 15 This is another possible consequence of structural and physiological changes in LM. 9 However, because it does not cause evident signs and symptoms, it becomes a situation of greater risk, which can cause chronic respiratory diseases. 15 There is a strong association between LM and silent aspiration, which raises the need to carry out more comprehensive swallowing assessments in patients with such alteration, since they will not always show signs of laryngotracheal aspiration. 9 In addition, it is known that silent aspiration has a high prevalence in children with some neurological impairment, 14 which raises the suspicion that the neuromuscular origin proposed for LM may also contribute to episodes of silent aspiration.

Currently, there are several techniques to evaluate, diagnose, and monitor patients with suspected swallowing disorders. Clinical swallowing assessments, instrumental assessments, and quality of life measures are the main tools used in the clinic. 16

The videofluoroscopic swallowing study (VFSS) is considered the gold standard for the assessment of swallowing and is the most reliable method for detecting silent aspiration. 9 However, due to its higher cost and the risks of radiation exposure, VFSS is only performed when aspiration is suspected, but it has not been possible to confirm it with the clinical evaluation of swallowing. As the VFSS is not a mandatory exam to assess swallowing, the chances of not detecting swallowing disorders are increased in some patients with LM, since many may have silent aspiration. 11 In turn, dysphagia and unidentified and managed aspirations increase the risk of compromising the airways, 9 15 influencing the functioning of the lungs, causing chronic lung diseases or even being fatal in cases of total airway obstruction. 16

The research started due to the need to identify and synthesize qualitative and quantitative data in relation to swallowing and the presence of changes in this process in children with LM, with or without the SGP surgical treatment, before and after it. Thus, these data would help to obtain an overview on the subject in order to contribute, later, in the clinical practice of care, diagnosis, and management of these patients. Thus, the objective of this systematic review is to identify the prevalence of oropharyngeal dysphagia in children with LM before and after SGP.

## Review of the Literature

The systematic literature review was conducted according to the instructions of the Cochrane Collaboration 17 and was reported according to the Preferred Reporting Items for Systematic Reviews and Meta-Analyzes (PRISMA) guidelines. 18 The study protocol was registered with PROSPERO - ( http://www.crd.york.ac.uk/PROSPERO/ ), under approval number CRD 42020175040.

The guiding question for the study was: “What is the prevalence of oropharyngeal dysphagia in children with LM before and after SGP?”. Subsequently, a search was conducted in the following electronic databases: PubMed, The Cochrane Central Register of Controlled Trials, Embase, Latin American and Caribbean Literature in Health Sciences (Lilacs), CidSaude, PAHO, IBECS, and Scielo. This research was complemented by a manual search of other bibliographic resources in the health area related to the presence of oropharyngeal dysphagia in patients with LM, in order to minimize selection bias. Then, studies published until April 2021 were included. For the search stage in the databases, the keywords were associated with specific medical subject headings (MeSH). The complete search strategy, with terms used for PubMed, can be seen in Table 1 . In addition, in order to increase the sensitivity of the search, entreterms and synonyms were included in the search strategy, which was adapted to the requirements of each database.

**Table 1:** Search strategy used in the PubMed database

**(#1) Patient**	*Infant* [Mesh] OR *Infants* OR *Infant* , *Newborn* [Mesh] OR *Infants* , *Newborn* OR *Newborn* *Infant* OR *Newborn* *Infants* OR *Newborns* OR *Newborn* OR *Neonate* OR *Neonates*
**(#2) Exposure**	*Tracheobronchomalacia* [MESH] OR *Tracheobronchomalacias* OR *Laryngomalacia* [MESH] OR *Laryngomalacias* OR *Chondromalacia* *of* *Larynx* OR *Larynx* *Chondromalacia* OR *Larynx* *Chondromalacias* OR *Cartilage* *Diseases* [MESH] OR *Cartilage* *Disease* OR *Chondromalacia* OR *Chondromalacias* OR *Laryngeal* *Diseases* [MESH] OR *Disease* , *Laryngeal* OR *Diseases* , *Laryngeal* OR *Laryngeal* *Disease* OR *Larynx* *Diseases* OR *Disease* , *Larynx* OR *Diseases* , *Larynx* OR *Larynx* *Disease* ” OR *Laryngeal* *Perichondritis* OR *Laryngeal* *Perichondritis* OR *Perichondritis* , Laryngeal OR Perichondritis, Laryngeal OR Bronchomalacia [MESH] OR *Bronchomalacias* OR *Chondromalacia* *of* *Bronchi* OR “Bronchi Chondromalacia OR *Bronchi* *Chondromalacias* ” OR *Tracheomalacia* [MESH] OR *Tracheomalacias* OR *Chondromalacia* *of* *Trachea* OR *Trachea* *Chondromalacia* OR *Trachea* *Chondromalacias*
**(#3) Outcome**	*RespiratoryAspiration* OR *Pneumonia* , *Aspiration* OR *Deglutition* *Disorders*
**Search**	***#1 AND #2 AND #3***

Only studies with an observational analytical design (cohort, case-control, cross-sectional, case study and case series) were included, without language or publication date restriction, with a population of children up to 24 months, of both sexes, diagnosed with LM, who presented clinical or objective swallowing assessment before and after SGP surgery. In this study, instead of interventions, exposures were analyzed, with LM being the exposure of interest. Oropharyngeal dysphagia was considered the main outcome of this review, identified through clinical and objective swallowing assessments, whose main signs are penetration and/or laryngotracheal aspiration.

The studies were initially evaluated by the title and abstracts by two independent evaluators. Studies that met the eligibility criteria were included, and all studies were listed as *included* , *excluded,* or *not clear* . The full texts of the studies included in this stage were obtained and independently assessed by the two reviewers. The reasons for exclusion of the full texts evaluated were recorded, and a third reviewer participated in the research to carry out possible bias between the articles that would be included or not. After the consensus or deliberation of the third reviewer, the articles included were transferred to the data extraction stage, using a standard form in Microsoft Excel (Microsoft Corp., Redmond, WA, USA). The following variables were extracted: authors, year of publication, methodological design of the study, total number of subjects and their characteristics (sex and average age), number of subjects with LM, number of subjects who underwent SGP, associated comorbidities, type of evaluation of swallowing performed, and data on swallowing disorders before and after SGP surgery, such as the frequency of dysphagia in the sample. Disagreement situations in the data extraction stage were solved by the third reviewer. For each result of interest, the number of participants in each group, baseline, and change in the mean (or median), standard deviations, interquartile, and baseline intervals (or standard errors, or confidence intervals), when present, were extracted.

The risk of bias was recorded for each study using quality assessment tools 19 specific for observational studies. The same was applied by two independent evaluators, and the strength of the evidence was classified by reference to the total positive results for the 14 criteria recommended in the tool. In the present investigation, studies with a *yes* answer to questions 7, 8, 9, 10, 11, and 14, or those with adequacy for at least 50% of the 14 items, were considered to have a lower risk of bias. From its application, it was identified that all articles presented a high risk of bias, presenting methodological weaknesses.

**Fig. 1:**
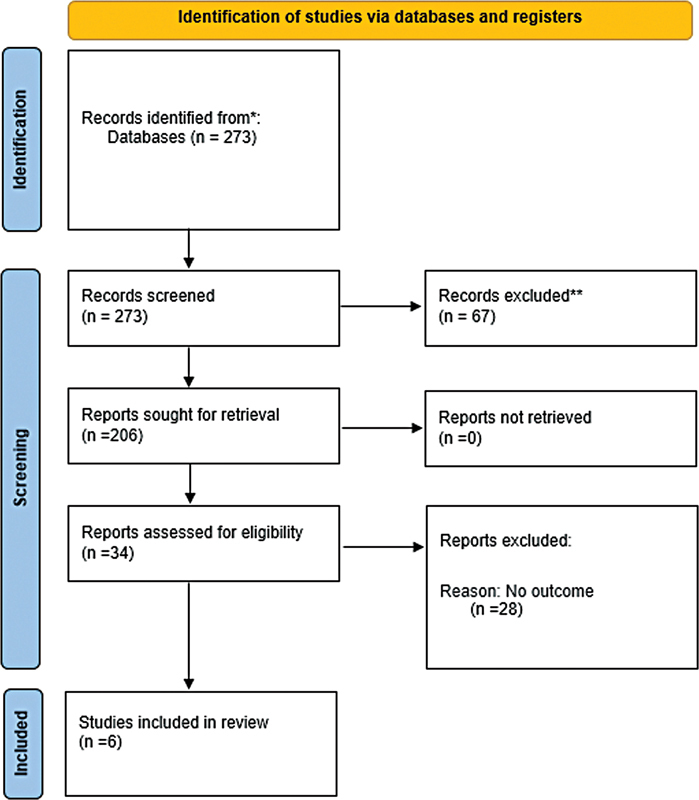
Study selection flowchart.

In the first stage of the article selection process in the databases, 273 articles were identified. After removing duplicates, the titles and abstracts of 206 records were analyzed. Of these, 34 references were selected for complete reading. At the end, 6 studies were included for qualitative analysis and, of these, 5 were selected for quantitative analysis - the case study was excluded. The flowchart of the entire process of inclusion and exclusion of studies is represented by Figure 1 . The characterization of the studies (authors, year of publication, study design, number of patients with LM, number of patients who underwent surgical correction of SGP, mean age, associated comorbidities, and types of swallowing evaluation) are in Table 2 . The works 20 21 22 23 24 25 were published between 2009 and 2019 and, for the most part, are cross-sectional articles. Together, they make up 330 patients with LM. Of these, 311 underwent SGP. The average age was 4 months. The types of swallowing evaluation performed were the clinical evaluation of swallowing, VFSS, and videoendoscopy of swallowing.

**Table 2:** Characteristics of the included studies

Study, year	Type of study	(n)* with LM*	(n)* with SGP	Average age (days)	Associated comorbidities	Assessment
Richter et al., 2009 20	Cross-sectional	50	50	136	GERD, congenital heart disease, neurological disease, genetic disease.	VED
Rastatter et al., 2010 21	Cross-sectional	39	39	48	Prematurity	VFSS
Powitzky et al., 2011 22	Cross-sectional	20	20	118.6	Translocation of chromosome 9 and 15, myasthenia gravis, down syndrome, tracheomalacia, micrognathia, macroglossia, glottic stenosis	VFSS
Durvasula et al., 2013 23	Cross-sectional	176	176	129.8	Tracheomalacia, bronchomalacia, laryngeal edema, unilateral vocal fold paralysis, subglottic stenosis, laryngeal cleft, prematurity	VFSS
Givens et al., 2018 24	Case Study	1	1	121	GERD, bronchiolitis, and pneumonia, cricopharyngeal achalasia	VFSS e VED
Scott et al., 2019 25	Cross-sectional	44	25	96	Down syndrome, genetic syndrome, neuromuscular disorder, prematurity, craniofacial anomaly, GERD, asthma, congenital anomaly of the heart and lung, anomaly of the upper airways.	VFSS

Abbreviations: GERD, gastroesophageal reflux disease; LM, laryngomalacia; n, number of subjects; SGP, supraglottoplasty; VED, swallowing videoendoscopy; VFSS, videofluoroscopic swallowing study.

mean age **: mean calculated based on the standard deviation offered by the article.

Table 3 shows the prevalence of presentation of dysphagia data before and after SGP, according to what was presented in each of the studies. Such data were used to perform meta-analysis, when possible. In all, 196 cases of pre SGP dysphagia and 62 cases after SGP surgery were identified in the studies.

**Table 3:** Prevalence of presentation of dysphagia data before and after supraglottoplasty surgery

Study, year	Dysphagia pre	Dysphagia post
Richter et al., 2009 20	44	8
Rastatter et al., 2010 21	10	21
Powitzky et al., 2011 22	7	9
Durvasula et al., 2013 23	109	22
Givens et al., 2018 24	1	0
Scott et al., 2019 25	25	2

**Fig. 2:**
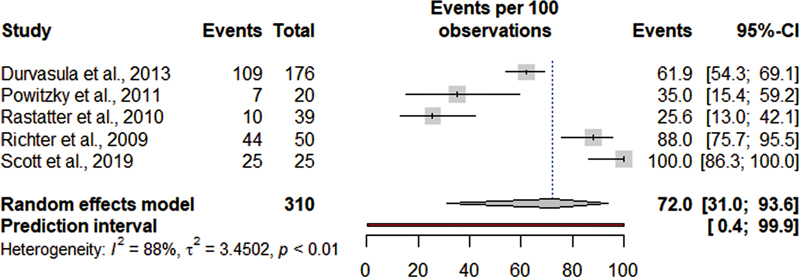
Meta-analysis of the frequency of dysphagia in children with laryngomalacia.

**Fig. 3:**
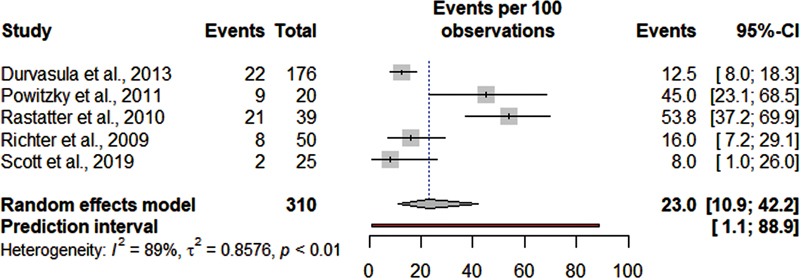
Meta-analysis of the frequency of dysphagia in children with laryngomalacia postsupraglottoplasty surgery.

Figure 2 shows the meta-analysis for the proportion of children with LM who presented dysphagia, which included 6 studies, 20 21 22 23 24 25 with a proportion of dysphagia in 72% of the patients, with a high heterogeneity (95% confidence interval [CI] = 31–93.6., I _2_ = 88%). In Figure 3 , a meta-analysis was presented for the proportion of children with LM and dysphagia after the SGP procedure, with a proportion of dysphagia being identified in 23% of patients, with a high heterogeneity (95% CI = 10.9–42., I _2_ = 89%).

**Fig. 4:**
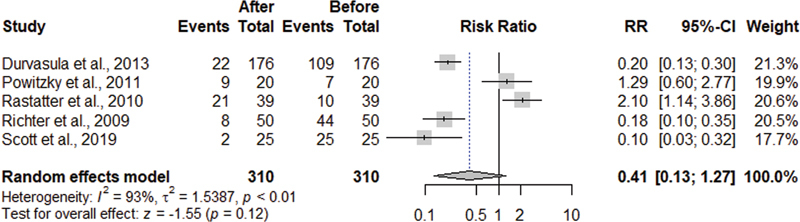
Dysphagia meta-analysis post versus presupraglottoplasty.

Figure 4 presents a meta-analysis with a random effect model on the effect of SGP in reducing oropharyngeal dysphagia in children with LM. The proportions of dysphagia pre and post SGP were compared, which included 6 articles. 20 21 22 23 24 25 This meta-analysis demonstrated that after SGP there was a 59% reduction in the prevalence of dysphagia with high heterogeneity (PR 0.41 [95% CI 0.13–1.27], I _2_ = 93%).

## Discussion

It is known that LM is one of the causes of swallowing disorders, both because it is an anatomical abnormality that affects the aerodigestive tract, and because it possibly compromises the coordination between suction/swallowing and breathing functions. However, there are still few studies with methodological rigor in order to identify the prevalence of such symptoms in the population of children with LM or the change in presentation after SGP surgery.

In this systematic review, an expressive frequency of children with LM who presented with dysphagia was identified—considered in this study as presentation of laryngotracheal penetration and/or aspiration. Also, that this frequency decreases after the SGP procedure is performed, and it may perhaps be directed as a procedure for children with LM and dysphagia.

The study by Powitzky et al. 22 presented a deviation in the meta-analysis, showing a higher occurrence of dysphagia in the postoperative period of SGP than patients presented preoperatively. This study evaluated the patients in the immediate postoperative period, and we considered this to be a possibility of bias, since these patients still have low sensitivity of oropharyngeal structures and decreased protective reflexes. 26 The authors also reported in their article that none of the 9 patients who presented dysphagia in the postoperative period continued to present dysphagia 1 month after SGP, suggesting that these children may experience transient dysphagia after the surgical procedure.

The study by Rasttater et al. 21 showed an increase in the frequency of oropharyngeal dysphagia in some patients after SGP. The non-establishment of time to perform the swallowing assessment is considered a determining factor since the study considered the VFSS from 24 to 48 hours after the procedure. Still, we consider that the increased prevalence of dysphagia in this study may be associated with the surgical techniques used for SGP, since this study evaluated two interventions cold steel SGP and CO _2_ laser SGP, which can be a determining factor for the difference between other studies in the literature.

The present study did not propose a comparison between surgical techniques for SGP and its impact on the reduction of dysphagia in children with laryngomalacia, which is one of the possibilities for future studies, since Rasttater et al. 21 presents the comparison between techniques with different impact in swallowing of children with LM.

As limitations of this work, it can be highlighted that many of the included studies did not present important data, such as the gender of the participants and methodological weaknesses. The selected articles are heterogeneous and differ in several points, such as homogeneity of the comorbidities associated with the study population, main outcomes analyzed, outcome measures used and their standardization, among others.

The high heterogeneity identified in the meta-analyses can be justified by the heterogeneity of the population with LM, also identified in clinical practice. They are children with other associated comorbidities, and the vast majority with GER, as reported in the literature. 2 4 It is relevant to show that homogenizing this sample would not represent the real findings of patients in hospital admissions, who have multiple comorbidities.

In cases in which swallowing assessment was performed after SGP, not all studies reported how long after the procedure the assessment was performed, which can also interfere with the findings, since surgical interventions, especially those that change the anatomophysiology of the organs and structures involved in the swallowing process, can modify, in turn, the swallowing dynamics, which can be a causative factor of dysphagia.

## Final Comments

Through the present study, it was possible to verify a high frequency of dysphagia in children up to 2 years of age with LM, and that this frequency is reduced after the SGP procedure. There was a 59% reduction in the presentation of dysphagia immediately after the procedure—with high heterogeneity.
